# Functions of Polycomb Proteins on Active Targets

**DOI:** 10.3390/epigenomes4030017

**Published:** 2020-08-17

**Authors:** Natalia Giner-Laguarda, Miguel Vidal

**Affiliations:** Department of Cellular and Molecular Biology, Centro de Investigaciones Biológicas Margarita Salas, Ramiro de Maeztu 9, 28040 Madrid, Spain; natalia.giner@cib.csic.es

**Keywords:** Polycomb, RING1B, active targets, enhancer, promoter, fine-tuning regulation, differentiation

## Abstract

Chromatin regulators of the Polycomb group of genes are well-known by their activities as transcriptional repressors. Characteristically, their presence at genomic sites occurs with specific histone modifications and sometimes high-order chromatin structures correlated with silencing of genes involved in cell differentiation. However, evidence gathered in recent years, on flies and mammals, shows that in addition to these sites, Polycomb products bind to a large number of active regulatory regions. Occupied sites include promoters and also intergenic regions, containing enhancers and super-enhancers. Contrasting with occupancies at repressed targets, characteristic histone modifications are low or undetectable. Functions on active targets are dual, restraining gene expression at some targets while promoting activity at others. Our aim here is to summarize the evidence available and discuss the convenience of broadening the scope of research to include Polycomb functions on active targets.

## 1. Introduction

The genetic information encoded in eukaryotic DNA is managed through the intertwined actions of a large collection of transcription factors and chromatin regulators. Eukaryotic genomes are organized as long fibers of repeated nucleosomal units made of DNA wrapped around an octameric set of histone proteins, an evolutionary successful structure serving gene expression and replication functions. Transcription factors confer the specificity to regulate transcription by recognizing DNA sequences with various affinities. On the other hand, chromatin regulators, usually present as multiprotein complexes, modulate the interactions of transcription factors and transcriptional machinery with the DNA template, usually restricted by nucleosomes [1]. Chromatin regulators encompass a large collection of proteins and multiprotein complexes responsible for the so-called epigenetic regulation of the genome. The modified chromatin, the epigenome, resulting from histone modifications, location and mobility of nucleosomes, or DNA methylation include marks that sometimes can be inherited throughout cell divisions. As a whole, these modifications depict cell type-specific epigenomic landscapes with predictive properties about the presence and activity of DNA elements essential in transcription regulation, such as enhancers and promoters. For example, nucleosomes decorated with mono- or trimethylated forms of lysine 4 of histone H3 locate to enhancers and promoters, respectively. Also, the state of lysine 27 of histone H3, that in acetylated or trimethylated form corresponds with active enhancers or repressed enhancers and promoters, respectively [2]. Transcriptional activity, determined by communication between enhancers and promoters, can be affected by the organization of chromatin in mutually exclusive compartments of similar transcriptional activity (A, active and B, repressed). In addition, at a smaller size-scale, topologically associated domains (TADs) contain sequences that interact preferentially between themselves and not with those of other domains and may contain loop-folded structures mediated by architectural proteins (reviewed in [3,4]).

Among chromatin regulators, the machinery of the Polycomb group (PcG) of proteins is present throughout most eukaryotic groups. Identified first during the genetic analysis of the development of the fly *Drosophila melanogaster* [5] soon it was associated with transcriptional repression of developmentally relevant genes as the homeotic genes, an activity conserved in vertebrates and plants (summarized in [6,7]). Thus, it was only natural that the first biochemical assemblies of Polycomb proteins were termed Polycomb repressive complexes (PRCs) [8,9,10,11,12]. Accordingly, in the prevalent models used in the study of the Polycomb system, *Drosophila* tissues and mammalian embryonic stem cells (ESCs), Polycomb proteins localize at promoters and other regions of silent loci that, generally, undergo derepression upon the depletion of Polycomb components [13,14]. The interest on the Polycomb system was significantly encouraged not only by the key roles in differentiation of stem and oligopotent cells but also by their involvement in malignant transformation processes [15,16]. Our reference to their functions as transcriptional repressors will be only in passing, to provide some background on the best-known activities of components. Detailed descriptions can be found in a number of reviews [6,17,18,19,20].

Instead, we will focus our attention on a number of observations that, accumulating steadily, link unquestionably the Polycomb system, or at least some of its components, to the modulation of transcriptionally active loci. Within the conventional understanding Polycomb repressive functions are antagonized by the Trithorax system (the products of the antagonistic Trithorax group of genes [21,22]), acting on common control regions as in a two-sided system. The activities we will address, instead, deal with functions compatible with gene activity that Polycomb products modulate negatively at some loci, and positively, potentiating gene expression, at others. Compared to studies on repressive functions, it is clear that research efforts invested in roles on active targets reflect the larger attention commanded by canonical Polycomb activities. The relevance of recent genetic analysis in flies and mice, showing active Polycomb targets as part of tumor suppressor functions and of differentiation programs, led us to believe that this aspect of Polycomb activity merits further work.

## 2. The Polycomb System

**Figure 1:**
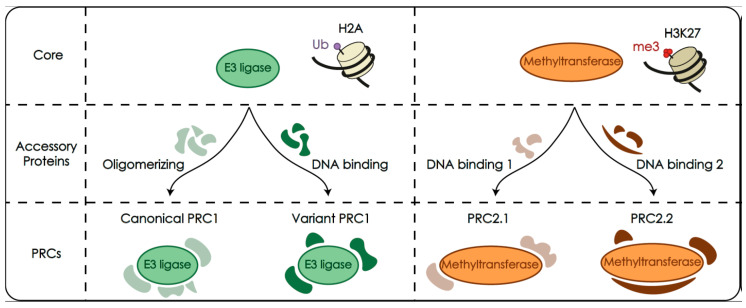
Simplified architecture of Polycomb repressive complexes (PRCs). Assemblies contain catalytic cores that monoubiquitylate histone H2A (green) or methylate histone H3K27 (orange). These cores correspond to heterodimeric RING E3 ligases, or to a multiprotein complex containing a lysine methyltransferase, respectively, and define the two large classes of Polycomb complexes, PRC1 and PRC2. The catalytic activities, recruitment to targets and other functions, are largely determined by the presence of a variety of specific accessory subunits that result in a heterogenous collection of complexes expressed in a cell-context dependent manner. Subunits with oligomerizing abilities and other protein–protein interactions form the canonical class of PRC1 complexes, whereas DNA-binding subunits are found among the variant class of PRC1. Similarly, specific, exclusive subunits with DNA-binding abilities and other functions define the PRC2.1 and PRC2.2 types of PRC2 complexes.

The following is the overview of the biochemistry and functionality of the Polycomb system, most of which derives from studies aimed at understanding its role as chromatin regulators in gene repression. Biochemically, the system comprises a heterogeneous collection of unrelated proteins, that can assemble in a highly diverse set of protein complexes (schematized in Figure 1). The subset of subunits/complexes mentioned below have been listed in Table 1 for reference and clarity.

### 2.1. Polycomb Complexes PRC1 and PRC2 as Histone Modifiers

The biochemical complexity of Polycomb assemblies can be reduced to two large classes defined by the type of histone modification they produce: one group is that of complexes that modify the C-terminal end of histone H2A (PRC1 complexes), and the other that of complexes that modify the N-terminal of histone H3 (PRC2 complexes). Polycomb-induced H2A modification results in the monoubiquitylation of (predominantly) lysine 119 (H2AK119Ub) in mammals (lysine 118 in *Drosophila*), and is catalyzed by heterodimeric RING E3 ligases present in PRC1 complexes [23]. The RING1 half of the oligomer (RING1A or its paralog RING1B, in mammals; Sce in *Drosophila*) interacts with the ubiquitin-conjugating component (E2 ligase), acting as substrate adapter, together with the PCGF partner, as a nucleosome recognition module for H2A modification [24,25]. PRC2 complexes, on the other hand, methylate histone H3 at its lysine 27 (H3K27me1, me2 and me3), in a reaction catalyzed by EZH2 and paralog EZH1, lysine methyl transferases that use S-adenosylmethionine as a donor of methyl groups [9,10,11,12]. Additional enzymatic activities, associated to a few PRC1 subunits, i.e., SUMO E3 ligase of CBX4 [26] or histone H3K36 demethylase of KDM2B [27] are of restricted impact or of unclear relevance in gene control [28].

PRC1 and PRC2 can act together and also independently of each other [29,30,31]. For a long time, it was accepted that PRC1 was epistatic to PRC2. The current understanding, however, is that they can engage in a feed forward interaction that reinforce each other activities [32] and that on new targets PRC1 activity may precede that of PRC2 [33].

### 2.2. PRC1 and PRC2 Are Made of a Catalytic Module and Associated Accessory Subunits

Following an architectural design found in other chromatin modifiers, the variety of Polycomb assemble around a (relatively) invariant core, specific for each of the two classes of complexes. For PRC1, the heterodimeric catalytic core is formed by combinations of a RING1 protein, either RING1A or RING1B, with one of six paralogs of a family of RING finger proteins (Polycomb group RING finger proteins, PCGF1 to PCGF6; *Drosophila* homologs: Psc, Su(z)2, l(3)73Ah). Given that the presence of one or another PCGF seems to influence the association of accessory subunits, six groups of PRC1 complexes are considered, named after the PCGF component: PRC1.1 to PRC1.6 [34]. Moreover, the nature of the accessory subunits confers key differences in the biochemical activities of the complexes, such as the ability to participate in interactions mediating high-order chromatin structures. Thus, the presence of one of five chromobox (CBX) paralogs (CBX2, CBX4, CBX6, CBX7, and CBX8 in mammals), homologs of Polycomb (Pc) in *Drosophila*, the founder member of the Polycomb system and one of three *Drosophila* polyhomeotic homologs, PHC1 to PHC3 in PRC1.2 and PRC1.4, sets them apart in a category of canonical PRC1 complexes (thus termed because the original Polycomb function associated with the first biochemical isolates). The remaining complexes are known as non-canonical or variant PRC1 and share a RYBP subunit, or its paralog YAF2 that interact directly with RING1 subunits in a mutually exclusive fashion with CBX proteins [35].

The catalytic core of PRC2 consists of the methyltransferase EZH1 or EZH2, together with two solenoid-shaped WD40 proteins, EED and RBB4 (or paralog RBBP7), and SUZ12 [E(z), ESC, CAF1 and Su(z)12 homologs in *Drosophila*, respectively]. This assembly is exquisitely sensitive to interactions, for example with modified histone tails, that influence allosterically EZH2 catalytic activity [36,37,38]. As before, the mutually exclusive association of accessory subunits to this holocomplex determines two main groups of PRC2 assemblies: PRC2.1, with PALI homologs (PALI1,2) and Polycomb-like (PCL) homologs, PCL1 to PCL3, or PRC2.2 with AEBP2 and JARID2 subunits [39,40,41].

In addition to the ability of CBX and PHC subunits of canonical PRC1 to engage in high-order chromatin structure [42,43,44,45], other important activities are contributed by accessory subunits. For example, binding to DNA KDM2B, MGA-MAX, or E2F6-DPA in PRC1 [46,47,48,49], PCL homologs, JARID2 or AEBP2 in PRC2 [50,51,52,53,54], or specific recognition of histone tails (CBX subunits in PRC1 or EED and RBBP4,7 in PRC2 [36,55,56]. The pool of Polycomb complexes displayed in each cell type is determined by the expression levels of each of the subunits, an aspect of the system still poorly characterized. A quantitative study of PRC1 and PRC2 complexes displayed in ESCs and in neural progenitors (NPCs) derived from them, illustrates this point. The study describes dramatic changes in levels of PRC2 core subunits or exchange of PCGF and CBX subunits in PRC1 [57].

### 2.3. Catalytic Modifications of Histone Tails

PRC1 and PRC2 modify their substrate histones both in a ubiquitous and targeted manner. PRC1 monoubiquitylates histone H2A globally at a very low level and then, at a higher density in a focused manner, restricted to cis-regulatory sites such as promoter proximal sequences [58,59]. E3 ligases of variant PRC1 complexes are constitutively active whereas those of canonical PRC1 complexes are in an autoinhibited form from which they are released upon appropriate interaction with the nucleosome [60]. The enhanced E3 ligase activity of variant PRC1 complexes is related, at least in part, to paralogs RYBP and YAF2, present in all of them [61,62].

H3K27 methylation can affect large extensions of the genome as mono- and, preferentially, dimethyl stages [63,64]. The additional methyl group in H3K27me3—the characteristic Polycomb modification associated to gene repression—is superbly regulated in a combination of the kinetic requirement of the reaction [65] and allosteric changes of the catalytic core after sensing specific histone tail modifications. For example, the interaction of EED with a previously trimethylated tail of H3K27 in an adjacent nucleosome greatly activates EZH2 [36,66]. An alternative pathway implies H2AK119Ub stimulating the catalytic activity of complexes containing AEBP2 [32], confirmed by the impact on the extent of H3K27me3 in cells expressing inert RING1 E3 ligases [67]. In contrast, if the nucleosomal environment contains H3K36me3 the activities of EZH2 and E3 ligases of PRC2 and PRC1 are inhibited [68,69].

Within PRC1, the best-known interaction with histone tails is the recognition of H3K27me3 by some CBX subunits (CBX7, CBX8) for instance in canonical PRC1 [70,71]. Also relevant, in the recognition of H2AUb by PRC1 subunits RYBP-YAF2 [62,72], or JARID2 in PRC2 [73]. Of course, Polycomb-induced modifications are always in a balance with antagonistic activities of enzymatic complexes that remove them. Lysine-specific histone demethylases KDM6A and B or a subset of diverse deubiquitinases such as BAP1, USP16, or MYSM1, effectively modulate the extent of histone changes induced by PRC2 and PRC1, respectively [74,75,76,77].

Polycomb-induced histone modifications characteristically correlate with transcriptionally repressed states. Their function and relative impact on gene expression are loci and context-dependent, as in bivalent promoters—not active but poised for expression—recently re-defined as bistable [78] or large silent domains [67,79]. Thus, in ESCs, histone H2A monoubiquitylation seems essential to gene repression [67,80], whereas in other cell types, PRC1-dependent repression can take place in the absence of H2AK119Ub [81]. Importantly, nucleosomes decorated with H3K27me3 and H2AUb contribute to their propagation, on the same sites, throughout cell division [62,82,83].

### 2.4. Recruitment of Polycomb Complexes to Chromatin

Targeted localization, or increased residence times of complexes on chromatin, favors enzymatic modifications and functions involving protein–protein interactions. In contrast, non-targeted collisions leading to more transient contacts are probably the origin of global histone modifications mentioned above. Targeted recruitment can be direct, through DNA-binding subunits in the complex or, indirect, through contacts with non-Polycomb DNA binding proteins.

Cis-regulatory modules that mediate Polycomb repression (the so-called Polycomb response elements or PRE) were first identified in transgenic assays in *Drosophila* [84]. PREs sequences are enriched in sites for DNA-binding proteins [85]. Of these, only pleiohomeotic—a zinc finger protein homolog of vertebrate YY1 [86]—is encoded by a *PcG* gene. Recruiting of PRC complexes to PREs, however, depends on the concurrent activity of a varied set of DNA-binding proteins [87,88,89]. YY1, which is not part of Polycomb complexes, has a controverted role in recruiting in mammals [90,91]. Instead, PRC1 and PRC2 subunits with DNA binding activity can mediate localization to targets, in particular to those associated to non-methylated CpG-rich sequences (CpG islands, CGIs), a peculiar signature associated to a large number of mammalian promoters. Examples of DNA binding proteins stably associated to Polycomb complexes are KDM2B in variant PRC1.1 [46,47,48] and PCL homologs in PRC2.2 [50,51,52], that use as DNA binding motifs a CXXC-type of Zn finger and a winged helix, respectively. Interactions with modified histones, as H3K27me3 recognition by PRC1, at one time considered a key recruiting mechanism, is now considered to contribute only partially to Polycomb targeting [33,92]. Recruitment involving binding to RNAs is rather controversial and currently under active investigation [93,94].

Recently, single-cell tracking in vivo of Polycomb subunits has shed new light about association of PRC1 and PRC2 to chromatin. The new observations describe highly dynamic systems in which at any given time, only a small proportion of the subunits are found on chromatin, with brief residence times, and low rates of occupancy [95,96]. The rather unexpected stage, contrast with the view inferred from aggregated cell populations, more static derived from methodologies such as chromatin immunoprecipitation (ChIP).

### 2.5. High Order Chromatin Structures Mediated by Polycomb

Polycomb-anchored loops documented in *Drosophila* [97,98] and in mammalian cells [99] often encompass developmentally relevant genes, and are formed during development in a dynamically, discontinuous manner [98]. Polycomb-dependent long range promoter–promoter and promoter–enhancer contacts described in ESCs involve the *Hox* clusters and other genes encoding important developmental regulators [100] but also other genomic sites contributing to the overall 3D structure of the genome [101]. In mammalian cells, Polycomb mediated contacts between distal genomic regions are cell type specific, readily observed in ESCs but not in differentiated or tumoral cell types [102]. These long-range contacts withstand removal of cohesins [102], the molecules involved in chromatin organization through DNA loop extrusion [103,104] and, therefore, represent an independent element of chromatin organization. Some of these structures, are stabilized by PHC paralogs and other canonical PRC1 subunits containing a SAM domain, endowed with oligomerizing properties [42,43]. The requirement of H2AUb, however is controversial [67,101]. Also, despite the apparent correlation with gene repression, these contacts are separable of gene activity [101,105] as also suggested by their association with transcriptionally active loci (see below, [106]).

The presence, in a few PRC1 subunits, of so-called internally disordered sequences, allows them to bring genomic sites together, in condensates that separate them from other nuclear compartments. These phase-separated liquid structures are involved in a variety of cell biology processes [107]. Regarding gene expression, these condensates have been associated to specific transcriptional states such as those at active super-enhancers [108,109] or heterochromatic domains [110,111]. Mammalian CBX2 and *Drosophila* PSC are two of these subunits able to form these condensates in vitro [45,112,113,114] although evidence for their functional involvement in vivo is still preliminar.

## 3. Polycomb and Transcriptionally Active Loci

PRC1 loss-of-function mutations in mouse embryos lead to the identification of unexpected Polycomb activities sustaining gene expression. Examples illustrating this situation include the decreased levels of *Hoxb1* mRNA in PCGF2-deficient embryos [115] or the downregulated mRNA-encoding homeobox protein Nkx2.5 in embryonic cardiomyocytes deficient in PHC1 [116]. Additionally, chromatin localization studies in flies and mice showed that the presence of Polycomb subunits does not always correlate with transcriptionally repressed states. Work with transgenic flies shows that at least certain PRE-containing templates can undergo transcription without eviction of Polycomb-bound proteins [89]. In natural scenarios, in *Drosophila* cell lines PRC1 subunits PC, PH, and PSC bind to *Abdominal-B*, one of the genes in the *Bithorax* complex (*BX-C*) of homeotic genes, regardless of transcriptional state [117]. Similarly, in *Drosophila* tissues, subunits from PRC2 and PhoRC complexes associate with *Ultrabithorax* (*Ubx*)—another gene in the *BX-C* complex—both in wing and halter imaginal discs where *Ubx* is active and inactive, respectively [118,119,120]. In mice, PRC1 subunits PHC1 and CBX2, but not RING1B locate to *Hoxb8* sites both in anterior (active) and caudal (repressed) tissues of the embryos [121].

Recently, systematic analysis of Polycomb occupancies in mammalian cells and in *Drosophila* tissues show their presence on a large number of active loci (summarized in Table 2). The data listed refer only to subunits known or suspected to act together with other Polycomb subunits. Arbitrarily, we separate these instances from activities of Polycomb subunits that act with non-Polycomb proteins in functions that may include transcriptional activation. Examples are EZH2 acting as a positive cofactor with Androgen receptor in a subset of prostate cancer cell lines [122] or CBX8 in a non-PRC1 complex in mammary epithelial tumor cells [123].

The products encoded by Polycomb active targets somehow deviate from the conventional understanding of Polycomb functionality. In this traditional perspective, active Polycomb targets include genes encoding metabolic functions, cell proliferation, signaling, and cytoskeletal functions [124]. In a simplified view of Polycomb functions, conventional repressive roles would impact programs for alternative cell lineages, while functions on active loci would further progress along ongoing differentiation processes.

Although speculative, because of the rather limited data, a number of features identifiable in Polycomb regulation of active targets include:

- Presence of PRC1 (invariable, RING1B in particular) and PRC2 subunits;
- Low/undetectable levels of H3k27me3 or H2AK119Ub modifications;
- Chromatin enrichment rates generally lower than at Polycomb-repressed domains;
- In differentiated cell types, the ratio of active to silent targets larger than in cells of fly embryos or ESCs;
- Enhancers and super-enhancers, in addition to promoters, among regulatory sites occupied by Polycomb products.

From a gene control perspective, the outcome of Polycomb regulation of active targets includes both negative and positive influences, rather than the steady negative role(s) when acting on silent targets. An additional function promoting gene activation can be identified in cell differentiation processes by which the Polycomb system is involved only during the turning on process, without further implication while the gene is active.

### 3.1. Polycomb Occupancy of Active Targets

Data available seem to suggest that PRC occupation of active sites (Table 2) seem to fall into two categories. In one PRC proteins locate predominantly at promoters as in ESCs, B-cells, fibroblasts, epidermal progenitors, or the erythroleukemic cell line K562 [125,127,130,135,138]. In the other one, occupied sites also contain a large number of intergenic and intragenic or distal sites, including enhancers and super-enhancers. Examples of this situation are found in *Drosophila* imaginal discs [106,137], murine neural progenitors [57], and human breast tumor cell lines [140]. It is possible, however, that the notion of distinctive patterns of PRC binding to active targets has to be revisited in the light that ChIP analysis, in some cases, may have missed low density occupancies at distal, intergenic sites that may have led to the over-representation of promoters among binding sites. For example, in K562 cells, while one report describes predominant PRC1 binding to promoters [135], a large number of super-enhancers are identified among RING1B bound sites in these cells by other report [140]. An interesting observation is the relatively high representation of cancer cells among cell types with Polycomb functions on active targets. It is possible that the number of normal cell types investigated so far is rather limited, while transformed types are found in abundance among tissue culture cell lines used in labs. However, the observation can be meaningful by itself, reflecting some property of cancer cells related to the differential number of repressed/active Polycomb targets in primitive cells and in differentiating cells indicated above. It is possible that the deregulated transcription programs, a feature of transformed cell types, and the associated transcriptional dependence on the expression of a number of regulators [141] makes them singularly suited cell contexts for positive functions of, for example RING1B, supporting gene activity.

**Figure 2:**
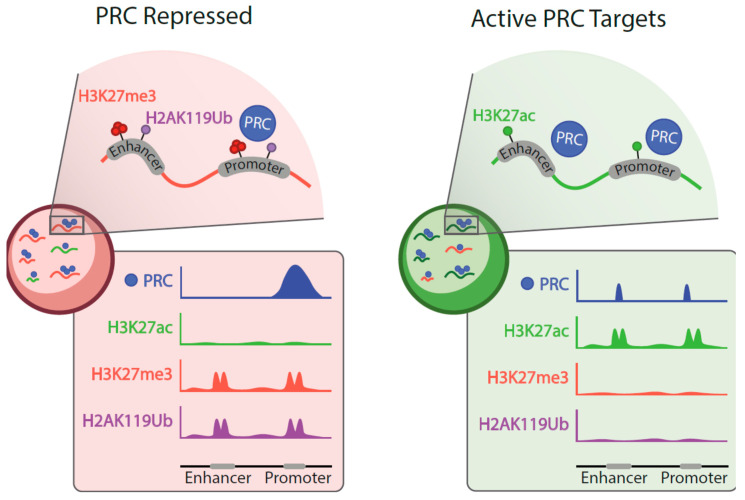
Schematized characterization of repressed and active Polycomb targets. Genomic sites occupied by PRC proteins in undifferentiated, primitive cells (**left**), and in cells in more advanced stages of differentiation pathways (**right**). Color-coded repressed and active targets show a higher proportion of active targets in differentiated cells. Polycomb-induced modifications are prevalent on repressed targets. Below are simplified views of ChIP profiles showing distinctive Polycomb occupancies (PRC, encompassing all classes of assemblies) and its increased presence on active enhancers (H3K27ac).

Few studies follow genomic localization of Polycomb products through differentiation [i.e., embryo to larva, ESCs or hematopoietic stem/progenitor (HSPCs) to their differentiated progenies]. If a trend was to be identified, it would be that the ratio of repressed to active Polycomb targets in primitive cells is high whereas, in contrast, in differentiated cells types the ratio is tilted toward the set of active targets [57,124,140]. During cell-state transitions, Polycomb is not only evicted from repressed sites but also displaced to new sites which are often active enhancers and promoters (Figure 2). Localization to the new active sites is probably mediated by transcription factors. For instance, in K562 cells, there is a significant overlap between promoter and enhancer occupancies of RING1B and the transcription factors GATA1 and GATA2 [140]. Likewise, in human leukemic cell line ME-1 there is a significant overlap between genomic sites occupied by RING1B and DNA-binding RUNX1 [142]. These examples support an instructive mechanism, mediated by transcription factors, in Polycomb recruiting to active targets. Furthermore, in breast tumor cell lines, sequences at RINGB-bound super-enhancers are enriched in ERα and FOXA1/2 consensus binding sites [140]. In this scenario, the molecular logic suggests that the pioneer factor FOXA1/2 makes room in nucleosome-wrapped DNA to facilitate the binding of ERα, with RING1B association somewhere along the process. However, such a straightforward view appears as a large oversimplification, as subsequent work shows binding of RING1B to estrogen response elements in response to hormone presence in a highly dynamic manner, together with FOXA1 and ERα, including mutual influences between the three proteins [143].

Binding patterns of Polycomb products to active sites partially differ from those of repressed domains (Figure 2). In general, occupancies extend not as broadly in active sites, and often ChIP peaks are sharp as those of transcription factors [140]. Also, occupancy densities, inferred from the reads counts are lower at active targets (see genome browser screen shots in [124,132,140]). These features, together with an idealized redeployment of Polycomb subunits in differentiated cell types are schematized in Figure 2.

Just as Polycomb is involved in the formation of loops and other contacts between occupied, distant sites in repressed domains, active Polycomb targets are tied in similar structures. In imaginal discs, a higher frequency of short- or long-range contacts correlates with the presence of PRC1 components on active promoters and enhancers [106]. These PRC1-tied loops, involving active targets form in a developmentally dynamic fashion. Similar chromatin arrangements in murine neural progenitors [106] indicates a conserved regulatory strategy, for both Polycomb-repressed and active targets.

### 3.2. Polycomb Proteins on Active Targets

ChIP experiments studying the localization of PRC1 often include the core subunit RING1B, and variably other subunits from canonical and variant PRC1 complexes. The presence of PRC2 proteins, in contrast, is much less studied. Polycomb-induced histone modifications, H3K27me3 and H2AK119Ub, usually found at repressed targets are, generally, absent. This is documented for *Drosophila* cell lines and imaginal discs [124,129] or in the murine system, in lymphoid [127], germinal [136], epidermal cells [138,139], hematopoietic progenitors, and their derived erythroid cells [132,144]. Polycomb-bound active targets in transformed mammalian cell lines also lack H3K27me3 and H2AK119Ub [135,140]. In contrast, many of these active targets are enriched in H3K27ac, a modification that together with H3K4me1 is typically associated to active enhancers [95,145]. Nevertheless, exceptions showing H3K27me3 on select active targets have been reported in neural and mesodermal progenitors derived from ESCs [128,133]. The effects of enforced decreases of H3K27me3, trough depletion of PRC2 products, shows minimal impact on active targets in imaginal discs or epidermal progenitors [124,138,139]. However, the wide down-regulation effect seen in EZH1-defficient erythroid cells, is unrelated to H3K27me3 because the prevalent PRC2 complex in these cells (lacking EED) is enzymatically inert [132].

The presence of H2AUb is generally undetectable or detected at low levels on active targets. The relatively little impact of this modification has been tested in epidermal progenitors depleted of RING1A and RING1B. The accompanying down regulation of active Polycomb targets, however, can be rescued to a large extent by an inert form of RING1B occupying these targets, thus showing that the transcriptionally positive influence of RING1B on these targets is independent of its E3 ligase activity [138]. Nevertheless, analysis in *Drosophila* imaginal discs, shows that chromatin at a subset of active enhancers associated to Polycomb targets is decorated with H2AK118Ub [106]. The observation probably indicates that there is more into the correlation between these histone modifications and transcriptional states than what can be anticipated at this time.

Although incomplete, the current evidence on the association of Polycomb proteins to active sites suggests that it may not occur, necessarily, as part of the known, biochemically defined complexes. In imaginal discs, for example, PH and PC bind to active regulatory elements independently of each other, in contrast with their colocalization at repressive PREs [137]. In ESCs, PCGF paralogs PCGF3 and PCGF5 act in gene activation functions independently of RING1A and RING1B [146]. These PCGF homologs bind their targets through interactions with DNA binding proteins outside the Polycomb realm, such as Tex10 or USF1/2 [146,147]. But even if occurring under canonical conditions, the functional outcome could be altered (i.e., reversed) by the proximity of interacting/non-interacting proteins able to modify the immediate molecular environment, making it conducive to gene expression.

**Figure 3:**
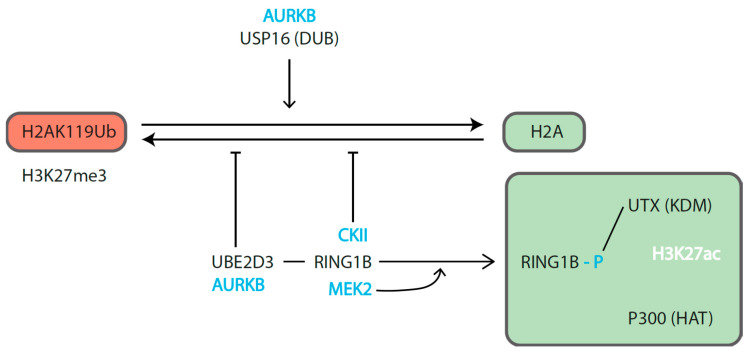
Posttranslational modifications of RING1B mediating opposite transcriptional states. Phosphorylation can accompany the activities of RING1B on repressed (**left**) and active (**right**) targets. The indicated kinases, in blue, correspond to reported examples where the outcome of its E3 catalytic activity is modulated directly (RING1B, UBE2D3) or indirectly (USP16). The continuous modification of H2A to oppose the action of DUBs, is needed to sustain the chromatin environment at PRC-repressed sites. Tilting the balance toward the non-ubiquitylated form of H2A may occur at the same time that RING1B phosphorylation (MEK2-induced) enhances its ability to associate with histone H3K27 demethylase (KDM) UTX-containing complexes. In turn, these facilitate the recruitment of histone acetyl transferase (HAT) p300, a well-known axis in enhancer activation.

Kinases are among the regulators that can flip RING1B roles (Figure 3). RING1B phosphorylation can inhibit its E3 ligase activity or affect its ability to associate direct or indirectly with proteins. For example, in resting peripheral B cells, Aurora B kinase (AURBK)—a kinase well known by its involvement in mitosis—indirectly inhibits RING1B E3 ligase activity through phosphorylation of ubiquitin-conjugating enzyme UBE2D3 [127]. At the same time, the deubiquitinase USP16 is phosphorylated to promote its deubiquitinase activity. The overall effect can be one of the efficient reduction of H2AK119Ub on promoter proximal nucleosomes. Direct down modulation of RING1B E3 ligase activity is also associated to its phosphorylation (at S168) by casein kinase II (CKII), a subunit regularly present as part of PRC1.5 complexes that in some cells include AUTS2, a subunit that can recruit histone acetylase (HAT) p300 [131]. Alternatively, posttranslational modifications of RING1B can affect its ability to associate direct or indirectly with proteins involved in transcriptional activation such as HAT p300 and histone H3K27 demethylases (KDM6A/UTX), as seen by MEK1 phosphorylation of RING1B S41 [134].

Not only RING1B phosphorylation can modulate its functions, but also the alteration in composition of Polycomb subunits is what affects the activity of the modified complex in other cases. This is the case mentioned above of PRC2 complexes in differentiating murine erythroid cells. There, a repressive, enzymatically active EZH2-EED-SUZ12 core complex in primitive cells, turns during differentiation into an inert EZH1-SUZ12 complex located on active targets [132]. The selective absence of PCGF2 from Polycomb-occupied promoters at active targets, while present at repressed sites of germ cells [136], maybe another example of how complex composition can dictate a stage conducive to gene expression.

Attempts to discern between contributions of canonical or variant PRC1 complexes are hampered by the evidence available nowadays, which is fragmentary and sometimes contradictory. For instance, in epidermal progenitors, there is a trend for the association of canonical subunits (PCGF4 and CBX8) with repressed targets and subunits from variant complexes, such as L3MBTL2, RYBP, or KDM2B, enriched on active sites [138]. In contrast, in mesodermal progenitors, some active targets are enriched in canonical PCGF2/CBX7 subunits and others in variant subunit RYBP [133]. Regardless of the PRC1 class, it appears that Polycomb assemble at active targets as combinations of PRC1 and/or PRC2 subunits, in a target-specific and cell-context dependent manner [57,132,133], compatible with functionality modifiable by associated/nearby non-Polycomb proteins.

### 3.3. Polycomb Functions on Active Genes

Polycomb proteins regulate active genes through mechanisms still not fully understood. For some targets a negative influence is observed (i.e., dampening activity), identified by the upregulation that follows depletion of Polycomb subunits [124,125,137]. This activity contrasts with the positive influence exerted on other targets (i.e., facilitating activity), which respond to Polycomb inactivation with decreased expression [124,125,137]. The outcomes of Polycomb inactivation/downregulation on active targets, however, are of a lesser magnitude than those usually observed when acting under its conventional repression function.

Evidence connecting Polycomb subunits with regulators associated to gene expression has been accumulating steadily. Thus, subunits of histone acetylase complexes MOF and Tip60 appear in the characterization of Polycomb interactors [148,149,150]. Cohesins, proteins that form ring-shaped complexes on promoters and enhancers [26] are interactors of PRC1 subunits [151] and, in *Drosophila*, assist their recruitment to active targets [129]. This biochemical knowledge, however, has not been integrated yet in a comprehensive mechanism underlying Polycomb functions on active genes.

#### 3.3.1. Dampening Gene Activity

The down modulation of active Polycomb targets is best documented in ESCs and in *Drosophila* imaginal discs. These studies are not readily comparable because of their inherently different resolution: single ESCs [152] and imaginal disc cell populations [124,137]. Nevertheless, active Polycomb targets differ in H3K27me3 modification, absent or very low in cells of imaginal discs. It is not clear whether the difference reflects distinct regulatory demands by the functional hardwiring of the targets, related to metabolic and signaling processes in ESCs [125], and to cell proliferation and polarity in imaginal discs [124]. Promoters, are occupied by the non-elongating, but active form of RNA polymerase II (RNApolII), which is phosphorylated at the serine 5 (S5P) in its C-terminal repeat domain at active targets, in contrast with the unphosphorylated, non-productive form of RNApolII of repressed targets [125,137,152].

Kinetic analysis of the activity of the Polycomb-bound active promoters in ESCs shows a lower frequency of transcriptional bursting than that of active genes, consistent with a switch between repressed and Polycomb-activated states [152]. These bursts represent the discontinuous nature of the general transcription process, so that the overall RNA output results of the combined effects of the size and frequency of bursts [153]. The highly variable expression per cell of Polycomb-bound active promoters could be due to Polycomb acting allelically, within every cell, or independently in every cell in the population, but it could not be determined because promoter activity was assessed as RNA level per cell [152]. The moderate increase in expression levels at Polycomb active targets in RING1A and RING1B-depleted ESCs has been correlated with a decrease in transcriptional noise, a parameter related to stochastic fluctuations in promoter activity.

**Figure 4:**
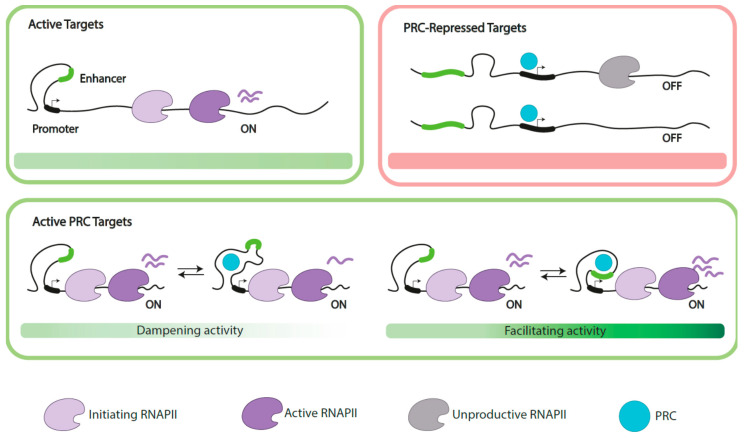
Simplified representation of cis regulatory elements at extended PRC targets. Depicted scenarios include active, not PRC-regulated targets (top, left), and PRC-repressed targets (top, right) as extreme scenarios, showing promoter proximal and elongating forms of productive RNA polymerases (RNAPII), in contrast with low/undetectable levels of mRNA at PRC-repressed targets, where RNAPII is in an unproductive form. Below, two possibilities of active PRC targets. In both cases, expression dampened (**left**) or facilitated (**right**) is interpreted as the outcome of the abilities of PRC proteins, together with uncharacterized factors, to modulate the communication between enhancers and promoters. Fine tuning of expression at these targets is illustrated as switching scenarios where PRC impairs such contacts to decrease overall promoter output (dampening activity), in contrast with the promotion of the contacts leading to sustained expression (facilitating activity). Target identities, accessory factors and available Polycomb products (cell context) are probable determinants of one or another function on these active targets.

Polycomb-bound sites at active targets are enriched among genomic three-dimensional contacts, although less extensively than Polycomb-repressed promoters [100]. Moreover, the proportion of active promoters that interact with active enhancers is significantly higher than that of repressed promoters [152], which are involved in contacts with poised enhancers [100,152]. Interestingly, since the communication of enhancers and promoters influences transcriptional bursting [110,154] it would be feasible that the intervention of Polycomb products in such communication becomes crucial for its regulatory role (diagrammed in Figure 4). Polycomb-dependent dampening of gene expression through control of transcriptional bursting may have been selected for the fine tuning of gene expression of selected targets in specific cell contexts.

The analysis of active Polycomb targets in imaginal disc cells, instead, enlightens dampening events related to the transition between paused (arrested shortly after initiated) and elongating transcription [137]. An idea of the unresolved complexity of the system is given by the different outcomes arising from the downregulation of one or another PRC1 subunit. Thus, while Ph acts against the presence of phosphorylated forms of RNApolII (both initiating, S5P, and elongating, S2P), SCE and PC promote their presence. Another marker of productive transcription that decreases with SCE depletion is SPT5, one of the two subunits of DSIF (DRB sensitivity-inducing factor, an essential complex for RNApolII release and productive elongation). These alterations take place at active, but not at repressed Polycomb targets. Of interest, the localization of SPT5 at enhancers and PREs also depends on SCE. In turn, the presence of PRC1 proteins on active targets is promoted by cohesins [129]. Additional work is needed to establish whether these findings apply to Polycomb-dampened targets in other cell types.

#### 3.3.2. Supporting Gene Expression

Examples of Polycomb-supported transcription are predominantly known in mammalian cells. Its identification comes from loss-of-function mutant experiments that results in decreased stationary mRNA and important alterations in differentiation programs of PRC1 mutant mouse lines. These experiments correlate with decreased expression of signature elements of cell lineage programs as is the case of those conducted on epidermis, germ cells, and early mesodermal cell types [127,133,136,138,139,155]. Cells where these targets have been identified, use Polycomb conventional-repressive activities in the silencing of alternate cell lineage programs, and Polycomb-promotion of gene expression in the progression of the ongoing differentiation.

The mechanism by which Polycomb supports transcription have not been elucidated yet. In general, depletion-associated gene downregulation is rather moderate, suggesting roles as positive cofactors. Following the same line of reasoning mentioned above, the influence on enhancer-promoter communication would serve to interpret Polycomb sustaining gene expression: by promoting communication; a higher activity at the promoter could be expected. Although there might be mechanistic differences, Polycomb subunits would be acting as coactivators. The accompanying histone modifications at regulatory sequences (i.e., H3K27ac enrichment) would buffer them against non-specific repressive influences. The fact that Polycomb subunits play a direct role sustaining gene activity is supported in epidermal progenitors by both the progressive decline in expression of active targets as a result of RING1A and RING1B depletion and the complementation of the defect by ectopic expression of RING1B [138].

The involvement of RING1B in the occurrence and maintenance of regulatory regions (assessed by Assay for Transposase-Accessible Chromatin (ATAC) experiments where accessible chromatin sites are evaluated through exposure to a transposase) has been documented in breast tumor cell lines [140]. The effects observed through manipulation of RING1B dosage are accompanied by changes in the activity of enhancers measured as variation in enhancer RNA (eRNA). Deregulation of active targets upon RING1B depletion varies with the tumor cell line, but in some cases, many undergo downregulation. Interestingly, chromatin accessibility alterations (gain and loss of ATAC peaks) associated to RING1B depletion occurs at enhancer and intergenic regions, but not at promoters [140]. Also, in cells with decreased levels of RING1B, variations in eRNA levels show both upregulation and downregulation of eRNAs, consistent with a dual role on enhancer function.

An entirely different approach, CRISPR-CAS mediated deletion of DNA sites occupied by PRC1, also demonstrates the involvement of PRC1 products in gene activation. These laborious experiments are loci-specific and do not involve changes in the levels of PRC1 products. In one case, the deletion of a promoter proximal site resulted in downregulation of the selector gene vestigial in the pouch of the wing imaginal disc, where it is normally expressed [156]. In a different study, deletions involve one or both of the PRC1-anchoring sites with contrasting effects on gene expression: while deletion of both sites decreases expression of *dac*, linked *CG588* gene is upregulated. Alternatively, deletion of the 3’ site decreases *CG588* expression with a much milder effect on *dac* [106]. In either manipulation, chromatin contacts are lost and hence is difficult to separate the contributions of the looped structure from that of the presence of PRC1 products.

#### 3.3.3. Indirect (Positive) Role in Gene Activation

In certain cases, gene expression induced during differentiation/development processes requires the activity of Polycomb proteins. However, its involvement seems to be limited to the establishment of the active transcriptional state rather than to its maintenance; as active transcriptional states are maintained without Polycomb presence.

**Figure 5:**
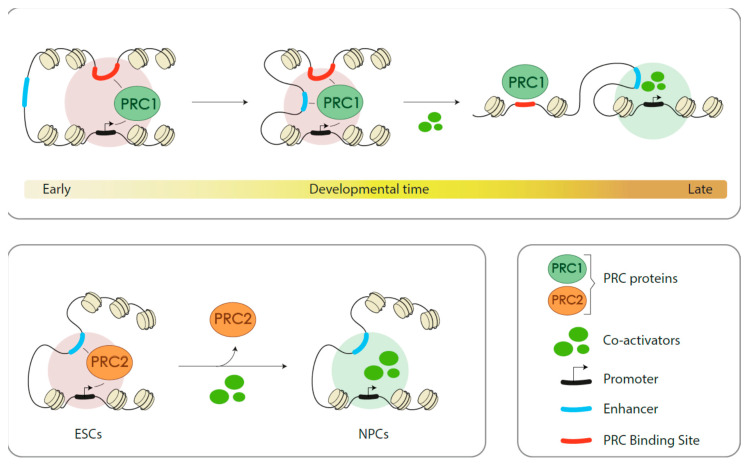
PRC topological activities are required for gene activation. Two situations are depicted, corresponding to reported cases where gene activation along differentiation pathways depends on the presence of PRC complexes. One, at the top, corresponds to configurations of the embryonic Meis2 locus throughout development [157]. Three-dimensional contacts, dependent on RING1B, evolve from the repressed state, left, where the enhancer cannot interact with the promoter, to the active state, where communication enhancer-promoter is effective, progressing through an intermediate state previous to the (assumed) involvement of DNA binding proteins and other factors required for gene activation. The second example, below, corresponds to configurations of one of the loci encoding anterior neural genes [158]. A major difference with the previous scenario is that, in the repressed state, in ESCs, enhancers (poised) are contacting the promoter, in a PRC2-dependent manner. The contacts as such, are not relevant to the transcriptional state, however, these are required for effective response to differentiating cues NPCs.

A well-studied example in mouse embryos is the spatiotemporal expression of *Meis2*; a developmentally important pleiotropic transcription factor. RING1B contributes positively (embryonic brain structures) and negatively (other tissues) to *Meis2* expression. RING1B binds constitutively a site located 3’ to the end of the locus and its occupation at the promoter varies with developmental time and expression states (Figure 5). Early in development, immuno-fluorescent in situ hybridation (immuno-FISH) analysis in non-expressing tissues yield close signals for the promoter and the 3’ binding site. These signals become distant in the derepressed state and are accompanied with the absence of RING1B [157]. Signals for the enhancer responsible of *Meis2* activation do not colocalize with those of RING1B-occupied sites. Later in development, this scenario changes so that, shortly before expression, signals for the three elements coalesce, in a RING1B-dependent manner. Subsequently, in the active site, signal proximity is observed only for promoter and enhancer [157]. The results are interpreted as RING1B playing a role in the communication between promoter and enhancer so that a chromatin conformation that prevents their interaction keeps silent *Meis2*, but that, later on, probably in cooperation with uncharacterized transcription factors, the eviction of RING1B from the promoter facilitates the interaction of the promoter with the enhancer inducing the activation of the *Meis2* gene.

Immuno-FISH lacks the resolution that can be acquired with Hi-C methods, but the correlation between overlapping signals and topological loops is widely accepted. Under this assumption, the structure proposed for the embryonic *Meis2* locus is similar to the PRC1-dependent contacts between promoters and enhancers in Polycomb repressed targets in ESCs [100]. However, their role in promoting the concomitant activation of these targets, while suggestive, remains to be studied. On the contrary, evidence along these lines has been shown, for PRC2-mediated chromatin structures associated to silent loci [158]. Here, contacts between promoters and poised enhancers are required for the induction of a subset of developmental regulators expressed in anterior domains of embryonic neural structures (Figure 5). The deletion of candidate enhancer sequences, using a CRISPR-Cas approach, impairs the contacts with the promoters of selected targets and results in poor expression in an in vitro differentiation system [158]. Inactivation of the core PRC2 EED subunit leads to similar results, although at the ESC stage, targets are not derepressed and the loss of H3K27me3 at poised enhancers is not accompanied by a gain of H3K27ac. This is probably due to the absence of the developmental signals required for neural activation in ESCs. This contrasts with the effects of PRC1 depletion on *HOX* clusters in ESCs, where promoter-promoter, but not promoter-enhancer contacts are lost upon RING1A and RING1B depletion, and poised enhancers become active and targets derepressed [100]. The overall impact of this strategy, turning a repressive Polycomb activity into one that facilitates gene induction, is not known.

## 4. Conclusions and Perspectives

Evidence for the activity of Polycomb proteins modulating the expression of active targets is incontrovertible. Nevertheless, it is a minor part of the current understanding about the Polycomb system, overwhelmed by the volume of work that supports functions on gene repression. How Polycomb proteins bind their active targets, probably not as the assemblies identified when acting in repressive functions, and how do they modulate transcriptional activity are areas in need of progress. Of particular interest is the finding of active enhancers among PRC1 sites. It is likely that mechanisms pertinent to the communication between regulatory elements, enhancer–promoter, are relevant to gaining insight in the role(s) of Polycomb proteins on active targets. It also underlines the relevance of the quantitative regulation of gene expression, in opposition to alternative on and off states. In this regard, Polycomb regulation of active genes is dual, either restricting transcription output, in some cases or, in others, supporting expression. Phenotypic consequences of defective regulation when dampening gene expression are not known. In contrast, decreased expression levels resulting from loss-of-function mutations are more easily correlated with impaired differentiation processes. It is clear that conventional approaches in the genetic analysis, downregulating/depleting any given subunit, lack the needed resolution. Instead, the use of allelic variants, as those being used to dissect catalytic from other activities, will be necessary.

Further studies are required to measure the impact of functions on active genes along cell lineage differentiation pathways. The current evidence, however, points at a large number of targets, including some important in the maintenance of cellular homeostasis. The detection of PRC1 products, RING1B in particular, on enhancers and super enhancer in tumor cell lines is intriguing. It appears, that as it has been previously observed for co-repressors [159], Polycomb, the system formerly known only as repressor, can handle additional identities in gene control.

## Figures and Tables

**Table 1:** Polycomb subunits mentioned in the text.

Complex	Mammals	Flies	Protein Motifs	Functions
PRC1 core subunits	RING1/RING1A, RNF2/RING1B	Sce	RING finger, RAWUL domain	RING1-PCGF pairs as heterodimeric E3 ligases that monoubiquitylate H2A
	PCGF1, PCGF2/MEL18, PCGF3, PCGF4/BMI1, PCGF5, PCGF6	Psc, Su(z)2, L(3)73Ah	RING finger, RAWUL domain	
canonical PRC1	CBX2, CBX4, CBX6 CBX7, CBX8	Pc	Chromobox	H3K27me3 recognition
	PHC1, PHC2, PHC3	Ph	SAM domain	Oligomerization, high order structures
variant PRC1	KDM2B	dKDM2	CXXC motif, jmjC, Fbox, LRR	DNA binding
	RYBP, YAF2	dRYBP	Zn finger	H2AUb recognition
	MGA-MAX	(1)		heterodimeric DNA binding module
	E2F-TFDP1	(1)		
	L3MBTL2	dSfmbt	MBT domains	
PRC2 core subunits	EZH1/EZH2	E(z)	SET domain	H3K27 methyltransferase
	SUZ12	Su(z)12	Several	Allosteric integration, recruitment
	EED	esc	WD repeats	H3K27me recognition
	RBBP4, RBBP7	Caf1	WD repeats	H3, H4 recognition
PRC2.1	PCL1, PCL2, PCL3	Pcl		DNA binding
	PALI1, PALI2, PALI3	(1)		Protein-protein interactions
PRC2.2	JARID2	Jarid2	JmjC, ARID	H2AUb recognition, recruitment
	AEBP2	Jing	Zn finger	DNA binding

(1) No PcG-related homolog identified.

**Table 2:** Polycomb subunits that localize (ChIP) to active targets.

PRC Subunits	Cell Type	Reference
RING1B, EZH2, SUZ12	Murine ESCs	[125,126]
RING1B, CBX7, EZH2	Murine quiescent B-cells	[127]
CBX8	Murine neural progenitors	[128]
Pc, PSc, Ph	*Drosophila* imaginal discs	[129]
CBX6, 7, 8; RING1A, RING1B	Human fibroblast cell lines	[130]
RING1B	Postnatal mouse brain cells	[131]
EZH1, EZH2, EED, SUZ12	Human differentiating erythroid cells	[132]
RING1B, PCGF2, CBX2, RYBP	Murine cardiac-mesoderm precursor cells	[133]
RING1B	Human melanoma cell lines	[134]
RING1B, PCGF2	Neural progenitors	[57]
RING1A, RING1B, CBX2, PCGF1, KDM2B	Human erythroleukemic K562 cell line, AML patient cells	[135]
Pc, Ph	*Drosophila* embryo, imaginal discs	[124]
RING1B, PCGF4	Human fibroblasts, K562 cells	[124]
RING1B, PCGF2	Murine spermatogonia cells	[136]
Pc, Ph	*Drosophila* BG3 cell line	[137]
RING1B, RYBP, PCGF4, KDM2B, L3MBTL2	Murine epidermal progenitors	[138,139]
RING1B	Human breast tumor cell lines MCF10A, T47D, MDA-MB-231; Human liver cancer cell line Hep G2, K562 cells	[140]
CBX4, PCGF2, PCGF4	Human breast tumor cell lines	[140,141]
RING1B	Human leukemic cell line ME-1	[142]
